# Increased risk of osteoporotic fractures and osteoporosis in patients with Addison's disease in Sweden: A nationwide population‐based cohort study

**DOI:** 10.1111/joim.20085

**Published:** 2025-04-06

**Authors:** Stavros Stergianos, Tim Spelman, Daniel Eriksson, Sara Öster, Sigridur Björnsdottir, Olle Kämpe, Jakob Skov, Sophie Bensing

**Affiliations:** ^1^ Department of Endocrinology Karolinska University Hospital Stockholm Sweden; ^2^ Department of Molecular Medicine and Surgery Karolinska Institutet Stockholm Sweden; ^3^ Department of Clinical Neuroscience Karolinska Institutet Stockholm Sweden; ^4^ Department of Medicine (Solna) Center for Molecular Medicine Karolinska Institutet Sweden; ^5^ Department of Immunology, Genetics and Pathology Uppsala University Uppsala Sweden; ^6^ Department of Medicine Karlstad Central Hospital Karlstad Sweden

**Keywords:** Addison's disease, adrenal insufficiency, fractures, glucocorticoids, mineralocorticoids, osteoporosis

## Abstract

**Background:**

The risk of major osteoporotic fractures (MOFs) and osteoporosis in patients with autoimmune Addison's disease (AAD) is unclear.

**Objective:**

To investigate the risk of MOF in patients with AAD and the possible correlation with adrenal hormone replacement doses.

**Methods:**

Swedish national health registers were used to identify 1869 subjects with AAD and 16,844 matched controls. The primary outcome was MOF, and the secondary outcome was treatment with osteoporosis medications. Marginal Cox models were used to compare time‐to‐event outcomes. The study period spanned from 1 July 2005 until 31 December 2020. Individuals at risk were followed from inclusion until censored or the end of the study period.

**Results:**

A total of 77 patients with AAD (7.1/1000 person‐years [PY]), and 387 matched controls (3.9/1000 PY) were diagnosed with MOF. The risk of MOF was higher in patients with AAD compared to matched controls, with an adjusted hazard ratio (aHR) of 1.82 (95% confidence interval [CI], 1.41–2.35) and increased in both male and female patients, with aHR of 2.51 (95% CI, 1.56–4.02) and 1.65 (95% CI, 1.22–2.24), respectively. Patients with AAD had an increased risk of treatment with osteoporosis medications: aHR 3.25 (95% CI, 2.71–3.99), compared to controls. No significant differences in MOF rates were observed between patients treated with intermediate or high doses of glucocorticoids compared to low doses (*p* = 0.967 and *p* = 0.580, respectively). Similarly, stratification by mineralocorticoid dose (<0.10 vs. ≥0.10 mg/day) showed no significant association regarding MOF (*p* = 0.915).

**Conclusions:**

The risk of MOF is increased in patients with AAD without any apparent correlation to adrenal hormone replacement doses.

## Graphical abstract

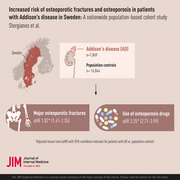

## Introduction

Supraphysiologic levels of glucocorticoids inhibit apoptosis of osteoclasts and promote apoptosis of osteoblasts, resulting in decreased bone formation [1]. Glucocorticoids also reduce intestinal absorption of calcium [2] and increase renal excretion of calcium by inhibiting its reabsorption in the renal tubule [3]. In addition, glucocorticoid excess inhibits the secretion of both growth hormone [4, 5] and gonadotropins [6]. Consequently, prolonged exposure to glucocorticoids, endogenous or exogenous, has detrimental effects on bone mineral density (BMD) and is the major cause of secondary osteoporosis [7, 8, 9, 10, 11, 12].

Autoimmune Addison's disease (AAD) is the major cause of primary adrenal insufficiency in industrialized countries [13]. It is caused by the autoimmune destruction of the adrenal cortex [14], which loses its ability to produce cortisol, aldosterone and androgens. Prevalence estimates vary between 5 and 221 cases per million, with the highest incidence observed in Europe and especially the Nordic countries [15]. AAD can occur as an isolated disease, but it is most commonly a major component of an autoimmune polyendocrine syndrome type 2 [16], frequently associated with autoimmune thyroid disease (∼50%), B12‐deficiency due to autoimmune gastritis, Type 1 diabetes mellitus (∼10%) [17], gonadal failure in women (6%–20% of women) [15, 18] and coeliac disease (2.3%–5.6%) [18, 19]. The standard treatment of AAD in Sweden is substitution with oral hydrocortisone two to three times daily with average doses of 28 mg/day [18]. Approximately 90% of patients receive mineralocorticoid replacement with oral fludrocortisone with a median dose of 0.1 mg daily [20]. Routine use of androgen replacement is not generally recommended [21, 22].

Current glucocorticoid replacement regimens in patients with adrenal insufficiency are not effective in reproducing the ultradian rhythmicity [23], and accurate assessment of dosing is cumbersome due to the lack of specific biochemical or clinical markers. Undertreatment with glucocorticoids is associated with symptoms of cortisol deficiency (fatigue, nausea, weakness), which usually resolve when an adequate dose is given, whereas moderate overtreatment with glucocorticoids has no apparent symptoms in the short term but detrimental effects in the long haul. This may contribute to an increased risk of overtreatment [24]. Indeed, according to the European Adrenal Insufficiency Registry, 21.4% of patients with primary adrenal failure received hydrocortisone doses of 30 mg/day or more [25]. Endogenous [7, 8, 12] and exogenous [9, 10, 11] glucocorticoid excess is associated with increased risk of osteoporosis and fractures; consequently, bone health in patients with AAD is of legitimate concern.

To our knowledge, there are no studies primarily focused on osteoporosis and its manifestations in patients with AAD. Small studies suggest that patients with AAD have lower BMD [26, 27, 28, 29], particularly when treated with high glucocorticoid replacement doses [29] or with synthetic glucocorticoids [13, 28]. The risk of vertebral [30] and hip [31] fractures is suggested to be higher in patients with AAD compared to controls. In other forms of adrenal insufficiency, impaired bone health has also been demonstrated. In a meta‐analysis, Li et al. found increased fracture rates in heterogeneous groups of patients receiving replacement therapy with glucocorticoids for primary or secondary causes of adrenal insufficiency [32]. Congenital adrenal hyperplasia (CAH) has also been associated with reduced BMD [33] and increased fracture risk [34]. Data regarding secondary adrenal insufficiency and bone health are scarce, with inconsistent findings as far as fracture risk is concerned [35, 36, 37]. Other autoimmune diseases commonly associated with AAD, such as Type 1 diabetes [38, 39] and Graves’ disease [40, 41, 42], are also associated with an increased risk of BMD reduction and fractures.

Overall, it is not clearly established if patients with AAD are at higher risk of developing osteoporotic fractures and osteoporosis, and if so, if the excess risk is attributable to replacement therapy, comorbidities and/or the disease itself. Moreover, it needs to be clarified if subgroups of patients are particularly susceptible to skeletal complications. The aim of this study was to investigate the risk of major osteoporotic fractures (MOFs) and osteoporosis in a large cohort of patients with AAD. Thus, we conducted a population‐based cohort–control study using an unbiased cohort representing the majority of Swedish patients with AAD by linking data from various Swedish national registers [43].

## Methods

This study was approved by the ethics review board of Stockholm, Sweden (DNR 2006/026/3 with several amendments, the latest DNR 2020–04374). The ethics committee did not require informed consent.

### Study population

This was a retrospective population‐based matched cohort study of patients and controls from the general population of Sweden. The Swedish personal identification number [44], assigned to all citizens upon birth or immigration, was used to identify study participants through linkage of several registers.

First, the National Patient Register, which dates back to 1964, with nationwide coverage since 1987, was used to identify individuals with a main or secondary diagnosis of primary adrenal insufficiency (PAI) or Addison crisis according to the international classification of diseases, Versions 7–10 (ICD‐7 274.4, ICD‐8 255.1, ICD‐9 255.E, ICD‐10 E27.1 and E27.2). The register includes data on hospital‐based inpatient care but also outpatient care since 2001. Diagnoses from primary care are not included.

Next, the Prescribed Drugs Register [20], in operation since 1 July 2005, was used to validate the diagnosis by requiring multiple (≥2) filled prescriptions of hydrocortisone or cortisone acetate (ATC H02AB09 or H02AB10) and multiple (≥2) filled prescriptions of fludrocortisone (ATC H02AA02) for inclusion. At this stage, the Swedish Population Register [43] was used to match each patient with 10 population controls for age, sex and county of residence at the time of the first ICD diagnosis of PAI or Addison crisis in the patient. The start of follow‐up was set to the day when the patient with AAD filled the second prescription of fludrocortisone or hydrocortisone/cortisone acetate for both patients with AAD and their matched controls. For the secondary outcome treatment with osteoporosis medications, the start of follow‐up was set to the earliest date of 1 January 2007 to exclude prevalent users of osteoporosis medications, as intravenous bisphosphonates are commonly prescribed and administered once per year.

At the start of the follow‐up, we applied exclusion criteria. First, to distinguish AAD from other forms of adrenal insufficiency, patients with diagnoses suggesting non‐autoimmune or secondary causes of adrenal insufficiency were subsequently excluded, along with their matched controls (Table S1). To avoid differences other than AAD between patients and controls, these criteria were applied to both patients and controls. Next, patients and controls with a diagnosis of MOF (≥18 years of age) before the start of follow‐up were excluded, along with all controls matched to patients fulfilling these exclusion criteria.

**Fig. 1:**
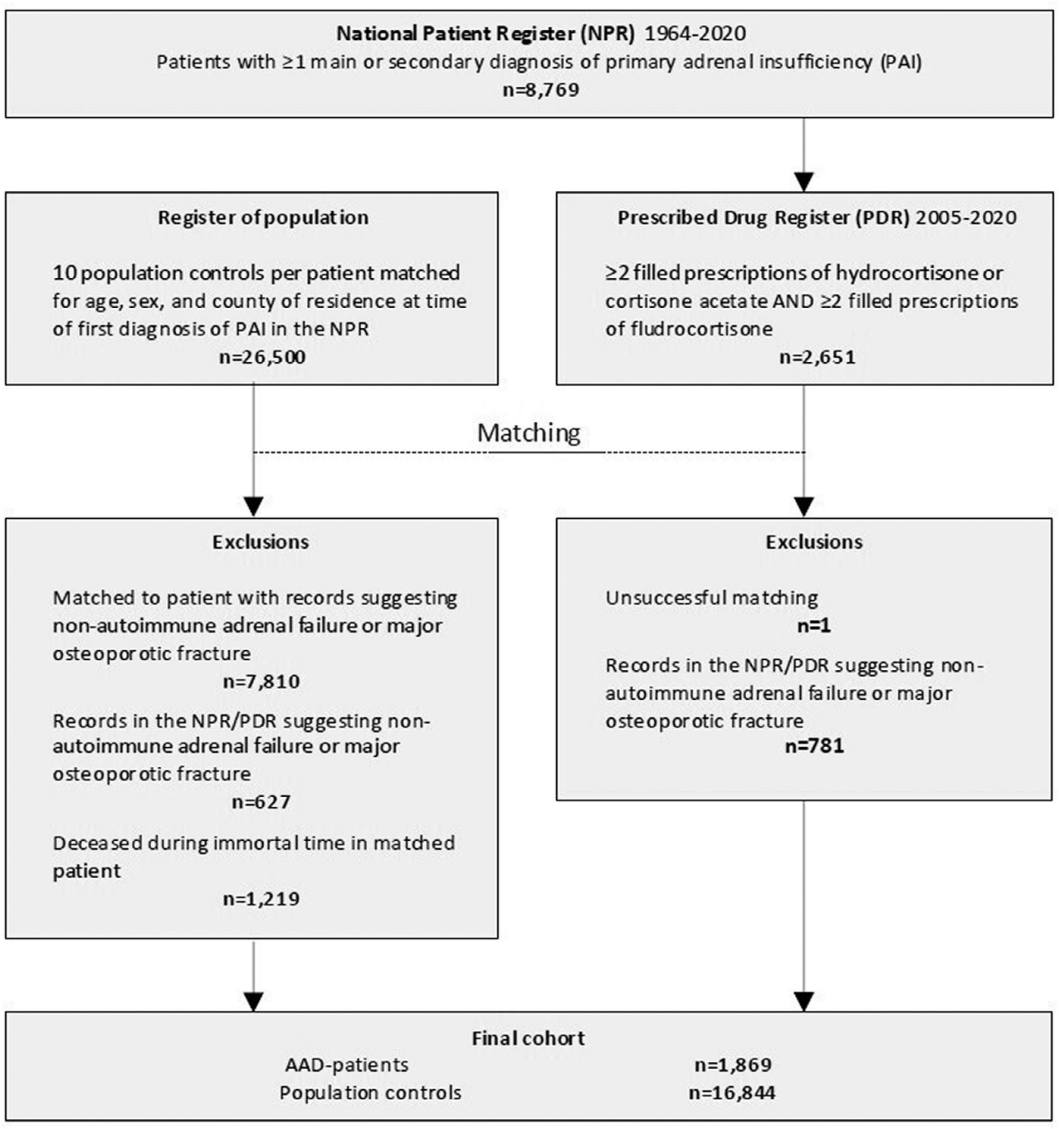
Flowchart of study inclusion. AAD, autoimmune Addison's disease.

Finally, controls who had died before the start of follow‐up were excluded. The steps followed during inclusion in the study are outlined in Fig. 1.

Follow‐up ended at the time of death, migration out of the country, manifestation of any MOF or end of the study period (31 December 2020), whichever occurred first.

### Outcome measures

The primary outcome of this study was MOF. MOF was defined as a fracture of the hip, distal antebrachium, vertebrae or humerus (Table S2). Treatment with osteoporosis medications was the secondary outcome of the study and included both antiresorptive and osteoanabolic agents (Table S3).

Subgroup analyses were conducted based on sex, age (<50 and ≥50 years) and duration of AAD at the start of follow‐up (<7, 7–<15 or ≥15 years). The age cut‐off was chosen because the average age of menopause in Sweden is 51 years. Moreover, it resulted in two strata of fairly similar size, which enabled subgroup analyses of acceptable precision. The latter applied also to the three strata of disease duration.

### Adrenal hormone replacement

Replacement doses were measured as time‐varying covariates (per year). To estimate the mean daily doses of hydrocortisone and fludrocortisone for each patient, we divided the total dispensed quantities (in mg) during follow‐up by the time of follow‐up (in days). Prescribed doses of hydrocortisone follow an approximately normal distribution, whereas those of fludrocortisone follow a bimodal distribution among Swedish patients with AAD [20]. Hence, the doses of hydrocortisone were stratified into three groups (<20, 20–30 and >30 mg), and doses of fludrocortisone into two groups (<0.10 and ≥0.10 mg). Cortisone acetate doses were converted into equivalent doses of hydrocortisone (dose of cortisone acetate/1.25 = dose of hydrocortisone). Synthetic steroids are rarely or not at all used in substitution therapy for AAD in Sweden.

### Comorbidities

At the start of follow‐up, we identified ICD‐10 codes for Type 1 and Type 2 diabetes, malignant neoplasms, thyrotoxicosis, ischaemic heart disease, cerebrovascular disease, chronic kidney disease, liver disease, rheumatoid arthritis, testicular hypofunction and primary ovarian failure. As a proxy for heavy smoking, the ICD‐10 code for chronic obstructive pulmonary disease (COPD) and/or the ATC‐code for drugs used in the treatment of nicotine dependence were used. As a proxy for high alcohol consumption, we used the ICD‐10 codes for alcohol‐related disorders and/or the ATC‐code for drugs used in the treatment of alcohol dependence (Table S4). The comorbidities adjusted for were chosen according to causes of secondary osteoporosis included in the Fracture Risk Assessment Tool (FRAX) [45] or other diseases associated with increased fracture risk.

### Sensitivity analyses

Several sensitivity analyses were conducted:

As vertebral fractures are often poorly diagnosed and documented in the national registers, a sensitivity analysis regarding non‐vertebral osteoporotic fractures (NVOFs) was conducted, defined as MOF with the exemption of vertebral fractures.

The influence of oestrogen on the risk of MOF was assessed by separately comparing rates of the primary outcome among patients with AAD and controls with (≥2 prescriptions) and without (<2 prescriptions) ongoing oestrogen use before the start of follow‐up.

In addition, sensitivity analyses were conducted on the risk of MOF excluding chronic users of prednisolone (≥2 prescriptions before the start of follow‐up or during the follow‐up period, ATC‐code: H02AB06) and individuals with a history of coeliac disease (ICD‐10: K90.0) before the start of follow‐up.

To assess whether antihypertensive treatment influenced the risk of MOF, the ATC‐codes C02, C03 and C07–C09 were used to identify individuals with one or more dispensations of antihypertensive drugs before the start of follow‐up. In a sensitivity analysis, these codes were included as an additional explanatory covariate in the multivariate model.

Finally, to assess the risk of MOF in incident AAD, we conducted a sensitivity analysis excluding patients with AAD (and their matched controls) with a first diagnosis of AAD prior to 1 July 2005. In this analysis, hydrocortisone and fludrocortisone doses were treated as time‐varying covariates. This was achieved by conducting serial measurements of mean daily doses for every drug dispensation (time from dispensation to next dispensation) and conducting separate Cox regressions for each specific time window before calculating a weighted average for all time windows.

### Statistical analysis

Categorical variables were summarized using frequency and percentage. Continuous variables were summarized using mean and standard deviation or median and interquartile range (IQR) as appropriate. Standardized differences were used to assess the balance of demographic and comorbidity factors between cases and controls in the matched sample. Univariable and multivariable marginal Cox models were used to compare time‐to‐event outcomes between cases and matched controls, adjusting for comorbidities and age at the start of follow‐up. For all time‐to‐event models, hazard proportionality was assessed via the analysis of scaled Schoenfeld residuals. Kaplan‐Meier survival and failure curves were used to visualize comparative time‐to‐event endpoints and a hazard proportionality test. For all analyses, *p* < 0.05 was considered significant. All analyses were undertaken in Stata version 17 (StataCorp. 2023. *Stata Statistical Software: Release 17*. College Station, TX: StataCorp LLC.).

## Results

A total of 1869 patients with AAD and 16,844 matched population controls fulfilled inclusion criteria (Fig. 1). Characteristics of study participants at the start of follow‐up are provided in Table 1.

**Table 1:** Characteristics and comorbidities associated with osteoporosis at the start of follow‐up in patients with autoimmune Addison's disease (AAD) and in matched controls.

Characteristics	AAD (*n* = 1869)	Controls (*n* = 16,844)	*p*‐value
Median age at baseline—years (IQR)	53 (38–68)	50 (37–63)	<0.001
Women—*n* (%)	1,014 (54.3)	9,109 (54.1)	0.885
Thyrotoxicosis—*n* (%)	100 (5.4)	128 (0.8)	<0.001
Testicular hypofunction—*n* (%)	9 (0.5)	7 (0.0)	<0.001
Primary ovarian failure—*n* (%)	16 (0.9)	4 (0.0)	<0.001
Type 1 diabetes mellitus—*n* (%)	266 (14.2)	168 (1.0)	<0.001
Type 2 diabetes mellitus—*n* (%)	169 (9.0)	464 (2.8)	<0.001
Rheumatoid arthritis—*n* (%)	28 (1.5)	139 (0.8)	0.003
Liver disease—*n* (%)	13 (0.7)	81 (0.5)	0.213
Alcohol‐related disorders—*n* (%)	24 (1.3)	429 (2.6)	0.001
Chronic kidney disease—*n* (%)	28 (1.5)	56 (0.3)	<0.001
Malignant neoplasms—*n* (%)	163 (8.7)	1,111 (6.6)	0.001
COPD—*n* (%)	66 (3.5)	444 (2.6)	0.024
Cerebrovascular disease—*n* (%)	56 (3.0)	388 (2.3)	0.062
Ischaemic heart disease—*n* (%)	54 (2.9)	457 (2.7)	0.658

*Note*: Age, sex and clinical characteristics associated with osteoporosis at the start of follow‐up.

Abbreviations: COPD, chronic obstructive pulmonary disease; IQR, interquartile range.

### MOF and treatment for osteoporosis

Study participants were followed for a total of 110,521 person‐years (PY) (10,832 in patients with AAD and 99,689 in matched controls). The median follow‐up time (IQR) was 4.89 (1.83–9.10) and 5.22 (1.63–9.64) years in patients with AAD and in their matched controls, respectively. Among patients with AAD 77 events (7.1/1000 PY) of the primary outcome (MOF) occurred, compared with 387 events (3.9/1000 PY) in the control group. MOF were significantly more common in individuals with AAD compared to matched controls, with an unadjusted hazard ratio (HR) of 1.87 (95% confidence interval [CI], 1.46–2.38), *p* < 0.001. Adjustment for comorbidities (Table 1) and age at the start of follow‐up returned an adjusted HR (aHR) of 1.82 (95% CI, 1.41–2.35), *p* < 0.001 (Table 2). The distribution of the first fracture by fracture type did not differ significantly between patients and controls (Table S5).

**Table 2:** Events, hazard ratios and adjusted hazard ratios for the primary, secondary outcome and non‐vertebral osteoporotic fractures in patients with autoimmune Addison's disease (AAD) and their matched controls.

	AAD (*n* = 1869)		Controls (*n* = 16,844)					
	Events, *n*	Events per 1000 PY (95% CI)	Events, *n*	Events per 1000 PY (95% CI)	HR (95% CI)	*p*‐value	aHR^a^ (95% CI)	*p*‐value
**Primary outcome**								
Major osteoporotic fractures^b^	77	7.1 (5.6–8.9)	387	3.9 (3.5–4.3)	1.87 (1.46–2.38)	<0.001	1.82 (1.41–2.35)	<0.001
**Sensitivity analysis**								
Non‐vertebral osteoporotic fractures^c^	74	6.8 (5.4–8.6)	363	3.6 (3.3–4.0)	1.77 (1.40–2.25)	<0.001	1.72 (1.33–2.23)	<0.001
**Secondary outcome**								
Medication for osteoporosis^d^	230	21.2 (18.6–24.2)	652	6.5 (6.0–7.1)	3.31 (2.83–3.88)	<0.001	3.25 (2.71–3.99)	<0.001

Abbreviations: aHR, adjusted hazard ratio; CI, confidence interval; HR, hazard ratio; PY, person‐years.

^a^ Adjusted for baseline comorbidities (Table 1) and age at start of follow‐up.

^b^ Fracture of the hip, distal antebrachium, vertebrae or humerus.

^c^ Fracture of the hip, distal antebrachium or humerus.

^d^ Antiresorptive and/or osteoanabolic drugs.

**Fig. 2:**
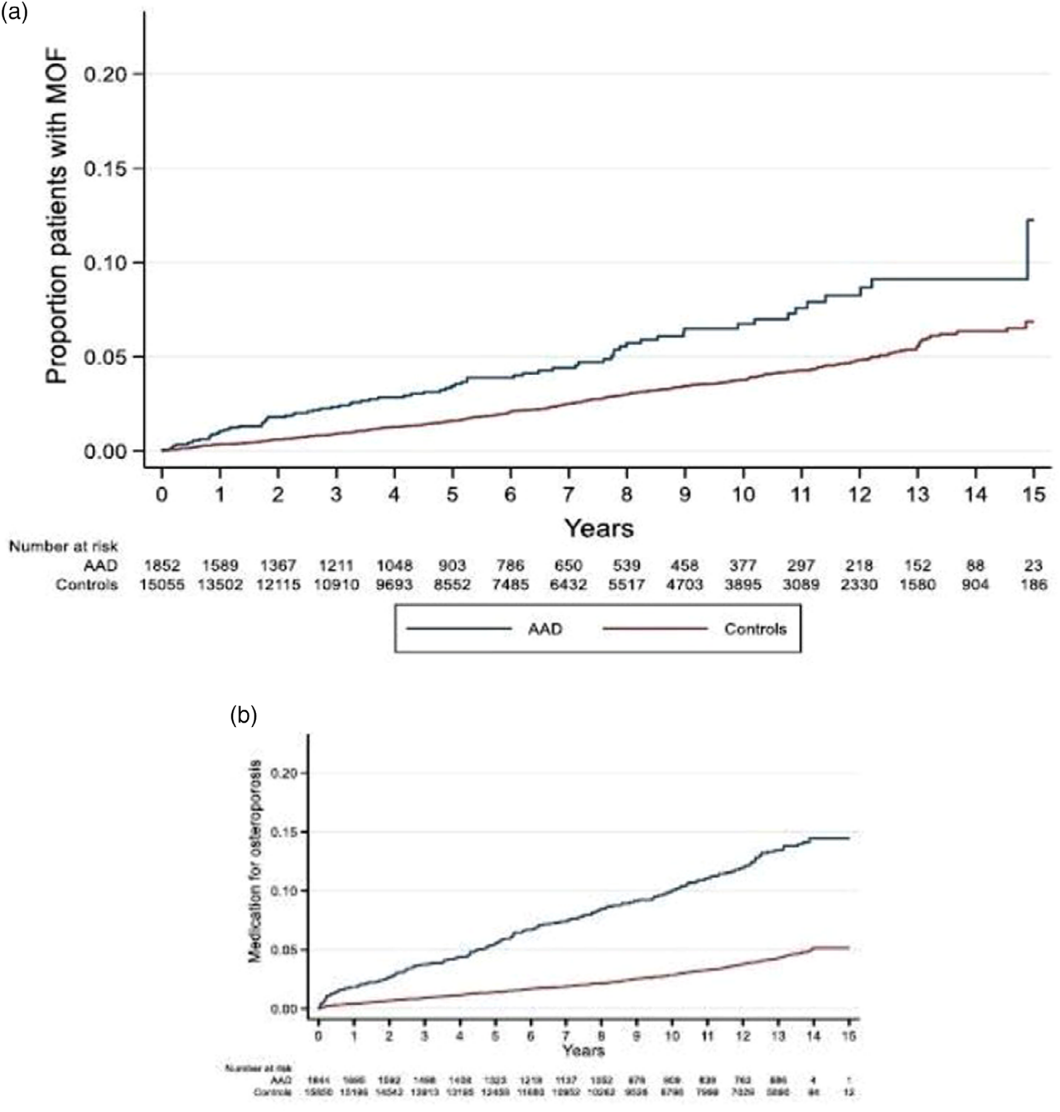
Cumulative proportion of patients with autoimmune Addison's disease (AAD) and their matched controls with primary (a) and secondary outcomes (b). Proportion of patients with AAD and controls experiencing major osteoporotic fractures (MOFs) (a) and treatment with osteoporosis medications (b). Note: Y‐axis truncated at 0.20.

Initiation of treatment with osteoporosis medications during follow‐up was more common in patients with AAD compared to their matched controls (aHR 3.25 [95% CI, 2.71–3.99], *p* < 0.001) (Table 2). The cumulative proportion of patients with AAD and controls experiencing primary and secondary outcome are illustrated in Fig. 2. The distribution of treatment categories (antiresorptive, osteoanabolic or other) are listed in Table S6.

### Subgroup analyses

**Fig. 3:**
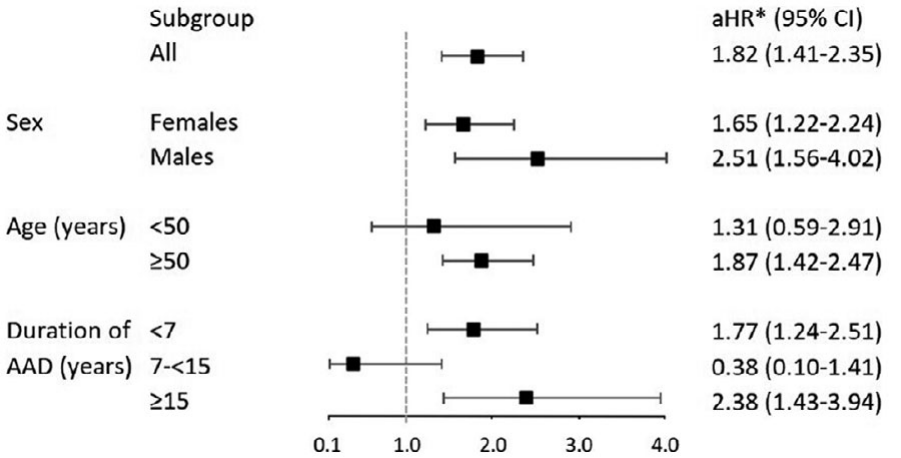
Primary outcome (major osteoporotic fractures [MOFs]) in subgroups of patients with autoimmune Addison's disease (AAD) and their matched controls. MOF in subgroups of sex, age and duration of AAD at the start of follow‐up. *Adjusted for comorbidities (Table 1) and age at the start of follow‐up. aHR, adjusted hazard ratio; CI, confidence intervals.

We examined the effect of sex, age (<50 and ≥50 years) and duration of AAD (<7, 7–<15 or ≥15 years) on the risk of MOF (Fig. 3). Both men and women with AAD had a significantly increased risk of MOF compared to their matched controls, with an aHR of 2.51 (95% CI, 1.56–4.02, *p* < 0.001) in men and 1.65 (95% CI, 1.22–2.24, *p* = 0.001) in women. The hazard rate in patients with AAD <50 years was not significantly elevated compared to controls, aHR 1.31 (95% CI, 0.59–2.91), *p* = 0.504. In contrast, patients ≥50 years had an increased aHR of 1.87 (95% CI, 1.42–2.47), *p* < 0.001. The rate of MOF was significantly increased in patients with a disease duration of <7 and ≥15 years but not in the subgroup of 7–<15 years. The highest rate was observed in patients with a disease duration ≥15 years (aHR 2.38 [95% CI, 1.43, 3.94], *p* = 0.001).

### Dose‐related outcomes

The median daily dose of hydrocortisone was 29.03 mg (IQR 22.18–33.10) (29.95 mg [IQR 25.42–34.45] in men and 26.51 mg [IQR 18.14–32.26] in women). The median dose of fludrocortisone was 0.09 mg (IQR 0.06–0.10), with similar doses in men and women. We observed no statistically significant differences in the risk of MOF with differing glucocorticoid or mineralocorticoid replacement doses (Table 3).

**Table 3:** Adjusted hazard ratios (aHR) for the primary outcome (major osteoporotic fractures [MOFs]) in patients with autoimmune Addison's disease (AAD) according to mean daily adrenal hormone replacement doses.

Drug	Category	AAD (*n*)	aHR^a^ (95% CI)	*p*‐value
Hydrocortisone^b^, mean daily dose (mg)	<20	411	Reference	
	20–30	756	0.99 (0.72–1.37)	0.967
	>30	702	1.09 (0.80–1.48)	0.580
Fludrocortisone, mean daily dose (mg)	<0.10	1217	Reference	
	≥0.10	652	1.01 (0.79–1.29)	0.915

Abbreviation: CI, confidence intervals.

^a^ Adjusted for comorbidities (Table 1) and age at start of follow‐up.

^b^ Cortisone acetate doses converted to hydrocortisone equivalent doses by dividing with 1.25.

### Sensitivity analyses

Subjects with AAD were more likely than controls to develop NVOF with an aHR of 1.72 (95% CI, 1.33–2.23), *p* < 0.001 (Table 2). This was evident in both men: aHR 2.60 (95% CI, 1.59–4.25), *p* < 0.001 and women: aHR 1.54 (95% CI, 1.15–2.07), *p* = 0.004.

The rate of MOF was significantly higher in women with AAD with <2 oestrogen dispensations before the start of follow‐up compared to controls with similar oestrogen status: aHR 1.82 (95% CI, 1.29–2.57), *p* = 0.001. It was, however, non‐significant among oestrogen users (≥2 dispensations before the start of follow‐up), aHR 1.44 (95% CI, 0.72–2.91), *p* = 0.302. The *p*‐value for interaction was non‐significant (*p* = 0.256) (Table S7).

Excluding study participants with ≥2 prescriptions of prednisolone (Table S8) or a history of coeliac disease (Table S9) before the start of follow‐up did not alter the risk of MOF.

Use of antihypertensives was significantly more common among patients with AAD (*n* = 636, 34%) compared to controls (*n* = 4542, 27%) (*p* < 0.001). The HR from this sensitivity analysis was almost identical to that of the primary analysis (Table S10), indicating that antihypertensive use did not affect the risk of MOF.

During the study period, we identified 893 patients with an incident diagnosis of AAD and 8572 matched controls. After adjustment for comorbidities, age at the start of follow‐up and time‐varying hydrocortisone and fludrocortisone doses, the aHR (2.18 [95% CI, 1.15–3.99], *p* = 0.011) remained similar to the main analysis. Adrenal hormone replacement doses did not significantly alter the risk (Table S11). The cumulative hazard plot for this sensitivity analysis is presented in Fig. S1.

## Discussion

In this population‐based cohort study, we demonstrate a higher incidence of MOF in patients with AAD compared to matched controls. This finding was evident in both men and women but more profound in men. No statistically significant correlation to adrenal hormone replacement doses was found.

Only two studies have assessed fracture risk in patients with AAD relative to matched controls. Björnsdottir et al. demonstrated in a Swedish epidemiological study an increased risk of hip fracture (HR 1.8; 95% CI 1.6–2.1; *p* < 0.001) [31]. In a prospective observational study, Camozzi et al. found a threefold higher prevalence of vertebral fractures in 87 Italian patients with AAD compared to matched controls [30]. Our results regarding both MOF and NVOF are congruous to those two studies but expand on previous findings. Notably, adjustment for potential confounders had marginal influence on HRs, suggesting AAD rather than comorbid conditions is the main cause of increased risk of osteoporotic fractures.

The increased risk of MOF was consistent across most subgroups and observed in both sexes, contrasting with findings on cardiovascular risk in AAD, which predominantly affects women [20]. Osteoporosis and osteoporosis‐related fractures are more common in women [46]. We were unable to find any dose‐related effects. The significance of treatment doses may be underestimated due to a lack of statistical power. Still, the high aHRs observed in individuals ≥50 years old and those with the longest duration of AAD (≥15 years), along with the absence of clear dose‐related effects, are consistent with an increased risk of MOF due to AAD, rather than excessive glucocorticoid exposure. The increased aHR in incident patients may imply that the negative impact of AAD on bone health is evident already in the early stages of the disease. This is in line with our finding of increased aHR of MOF in patients with short disease duration (<7 years) and results from Björnsdottir et al. showing increased risk of hip fracture in the first year after diagnosis [31]. Furthermore, the reliability of our findings was strengthened by conducting multiple sensitivity analyses on patients with prevalent and incident AAD, using different approaches to assess adrenal hormone dose effects.

Previous studies on skeletal health in AAD have mainly focused on BMD, yielding mixed findings. Results from older studies, when glucocorticoid replacement doses were generally higher, must be interpreted with caution. Nevertheless, some studies reported normal BMD in patients with AAD [28, 29, 47, 48]. This is exemplified by a study by Koetz et al. on 81 patients with PAI and 41 patients with CAH. The authors found that (low dose) glucocorticoid replacement was associated with BMD within the normal reference range [28]. In contrast, other studies have reported reduced BMD, predominantly in the lumbar spine [26, 27, 49, 50, 51]. The largest one by Løvås et al. found reduced BMD at the lumbar spine and the femoral neck in 292 patients with Addison's disease from Norway, New Zealand and the United Kingdom [27]. A BMD *T*‐score of −2.5 or less is consistent with osteoporosis, according to the definition adopted by the WHO [52]. Hence, the higher frequency of prescriptions of medications for osteoporosis, found in this study in patients with AAD compared to matched controls, is a reliable proxy for the diagnosis of osteoporosis, which is consistent with previous reports of reduced BMD in AAD. Osteoporosis treatment is designed to reduce fracture risk. Nevertheless, our study found that patients with AAD experienced a higher risk of MOF despite receiving intensified pharmacotherapy for osteoporosis.

Treatment with prednisolone [28, 47] or higher doses of glucocorticoids has been associated with a BMD reduction [29] in patients with PAI. Treatment of adrenal insufficiency with synthetic glucocorticoids has also been associated with adverse metabolic effects [53]. Other pathways may be involved, as dexamethasone is able to activate not only glucocorticoid receptors but also pregnane X receptors and progesterone receptors [54, 55]. Consequently, the latest guidelines from the Endocrine Society recommend replacement with hydrocortisone or cortisone acetate instead of long‐acting options [56]. There is currently no consensus on a threshold level below which glucocorticoid exposure has no negative impact on the skeleton. Traditionally, treatment with prednisolone has been divided into low (<2.5 mg daily, equivalent to <10 mg hydrocortisone), medium (2.5–7.5 mg daily, equivalent to 10–30 mg hydrocortisone) and high doses (>7.5 mg daily, equivalent to >30 mg hydrocortisone) [57]. In FRAX, glucocorticoid treatment with an equivalent dose of >5 mg prednisolone daily for 3 months or more is considered a clinical risk factor linked to increased fracture risk [45]. Recent data consider daily treatment with prednisolone doses as low as 2.5 mg as detrimental [58]. In line with this observation, Schultz et al. observed a significant increase in BMD in 27 patients (13 with PAI and 14 with CAH) after reducing the hydrocortisone equivalent dose from 30.8 ± 8.5 to 21.4 ± 7.2 mg [29]. We did not examine any dose‐related effects on the risk of the secondary outcome of diagnosis of osteoporosis, but our analyses were restricted to MOF. Surprisingly, we did not find a statistically significant risk increase for MOF in patients treated using medium (20–30 mg/day) or high (>30 mg/day) hydrocortisone replacement doses compared to low‐dose users (<20 mg/day). Because synthetic steroids are rarely used for substitution therapy in Sweden, any risk differences may be confined to these drugs (hydrocortisone and cortisone acetate) only. Nevertheless, this does not imply that the impact of hydrocortisone dosing is negligible, and a larger study sample may well have yielded statistically significant (but likely modest) dose‐related risks.

The results of this study showed no significant association between different replacement doses of mineralocorticoids and MOF. In a recent meta‐analysis [59], primary aldosteronism was associated with an increased fracture risk. Both osteoclasts and osteoblasts express mineralocorticoid receptors [60]. The mechanism through which excess aldosterone leads to poor bone health is, however, not fully understood [59]. Treatment of aldosterone deficiency (as opposed to mineralocorticoid excess) might be beneficial. Camozzi et al. found higher BMD in cortical bone in patients with AAD who were treated with fludrocortisone [30].

### Strengths and limitations

The major strengths of this study were its population‐based cohort design with prospectively collected data, a well‐defined and large cohort of patients with AAD, and a long study period yielding a considerable number of outcome events. Access to extensive data on comorbidities also allowed for relevant statistical adjustments, and the homogeneity of the results of subgroup and sensitivity analyses are indicators of high internal as well as external validity.

A limitation of this study was the lack of information on some variables associated with bone health, such as weight and height, physical activity and dual‐energy x‐ray absorptiometry (DXA) data. Proxies for heavy smoking and high alcohol consumption were used. Calculations of mean daily doses of adrenal hormones did not account for all variations in doses and were restricted to years covered by the Prescribed Drugs Register, that is, 2005 onwards. Thus, for patients with a diagnosis of AAD before 2005, dose estimates may not have been representative for the full duration of disease. In addition, doses might also have been slightly overestimated because the calculations are based on the dispensed and not consumed tablets. The secondary outcome in this study is sensitive to surveillance bias, given that patients with AAD, unlike most matched population controls, have regular follow‐ups in specialist clinics and are more likely to undergo DXA scans. This may have inflated HRs, but the high cumulative risks of treatment for osteoporosis observed in patients with AAD are still notable. The primary outcome is less sensitive to surveillance bias, and NVOF is unlikely to be affected at all. The validity of the National Patient Register is, in general, high [61], but it does not include diagnoses set in the primary care. Consequently, patients with AAD who are typically followed in medical outpatient care would be more likely to have a diagnosis of osteoporosis recorded in the National Patient Register than matched population controls receiving a corresponding diagnosis in primary health care, leading to detection bias. For that reason, we did not include the ICD codes for the diagnosis of osteoporosis in the study outcomes. This limitation does not apply to the Prescribed Drugs Register, which is used to retrieve information on prescribed medications, including primary care. The effect of treatment with androgens was not included in our analyses. Finally, we restricted our analyses to incident fractures. Considering that osteoporotic fractures are strong risk factors for repeat fractures, this limits our ability to assess the full impact of osteoporosis. This is, however, an interesting direction for future research, allowing for the assessment of both the risk of recurrent fractures and the impact of timely diagnosis and treatment of osteoporosis.

### Clinical implications

The findings of this study demonstrate that fragility fractures are more common in patients with AAD compared to population controls and that osteoporosis is common in this patient group. In the last version of the clinical guidelines of the Endocrine Society [56], there are no comments on the need for surveillance of bone health, but in our opinion, it should be an essential part of the monitoring of patients. Glucocorticoid replacement doses alone do not appear to explain this risk increase. Current treatment regimens, which, irrespective of dosing, do not adequately mimic the endogenous cortisol rhythmicity or other unknown causes related to the disease itself, might be possible contributing factors. Regular screening and treatment are needed to decrease the devastating effects of clinical manifestations of osteoporosis over time.

## Conclusion

Patients with AAD face a higher risk of sustaining MOF and receiving osteoporosis treatment compared to the general population. Regular surveillance and preventive interventions are motivated. We cannot show that current treatment regimens substantially explain the increase in risk. Further research on other possible underlying causes and mechanisms involved is needed.

## Author contributions

**Stavros Stergianos**: Conceptualization; writing—original draft; writing—review and editing; methodology; visualization; investigation; validation; project administration. **Tim Spelman**: Conceptualization; writing—original draft; software; writing—review and editing; methodology; formal analysis; visualization; investigation; validation. **Daniel Eriksson**: Writing—review and editing; data curation. **Sara Öster**: Writing—review and editing; data curation. **Sigridur Björnsdottir**: Writing—review and editing; methodology. **Olle Kämpe**: Funding acquisition; writing—review and editing; resources; validation. **Jakob Skov**: Conceptualization; writing—original draft; writing—review and editing; supervision; methodology; visualization; data curation; investigation; validation; project administration. **Sophie Bensing**: Conceptualization; funding acquisition; writing—review and editing; supervision; methodology; visualization; data curation; investigation; validation; resources; project administration; writing—original draft.

## Conflict of interest statement

S.S. is a site investigator with research funding to the institution from Kyowa Kirin and Takeda, both not related to this project. T.S. has received compensation from Biogen for serving on advisory boards, scientific leadership committees and steering committees. O.K. is a board member of BioCistronix AB and serves as chair of the Nobel Committee for the Physiology or Medicine Prize. D.E., S.Ö., S.B., J.S. and S.Ben. have nothing to declare.

## Supporting information




**Table S1**: Exclusion codes (applied to individuals with AAD and their matched controls).
**Table S2**: ICD‐codes for fractures (applied to individuals with AAD and their matched controls).
**Table S3**: ATC‐codes prescriptions for treatment of osteoporosis (applied to individuals with AAD and their matched controls).
**Table S4**: ICD‐10 codes of comorbidities related to increased fracture risk (applied to individuals with AAD and their matched controls).
**Table S5**: Incident events of first major osteoporotic fracture among study participants.
**Table S6**: Events of treatment with osteoporosis medication during the study period among study participants.
**Table S7**: Sensitivity analysis on the effect of subgroups of oestrogen treatment (in women only) on the risk of MOF (applied to both individuals with AAD and controls).
**Table S8**: Sensitivity analysis on the risk of MOF excluding individuals (patients with AAD and their matched controls) with ≥ 2 prescriptions of prednisolone (ATC‐code: H02AB06) before the start of followup.
**Table S9**: Sensitivity analysis on the risk of MOF excluding individuals (patients with AAD and their matched controls) with a history of coeliac disease* before the start of follow‐up.
**Table S10**: Sensitivity analysis on the risk of MOF adjusting for at least one dispensation of antihypertensive drugs* before the start of follow#x02010;up.
**Table S11**: Sensitivity analysis on the risk of MOF for patients with incident diagnosis of AAD and their matched controls.
**Figure S1**: Cumulative hazard plot of incident patients with AAD and their matched controls (incident cohort) with MOF

## Data Availability

The data that support the findings of this study are available on request from the corresponding author. The data are not publicly available due to privacy or ethical restrictions.
