# Supplementary material for: Increased risk of osteoporotic fractures and osteoporosis in patients with Addison's disease in Sweden: A nationwide population‐based cohort study
Source: J Intern Med. 2025 Apr 6;297(5):518–31. doi: 10.1111/joim.20085 (PMC12033000; doi:10.1111/joim.20085)
Supplement: Supplementary file 1 — Table S1: Exclusion codes (applied to individuals with AAD and their matched controls). Table S2: ICD‐codes for fractures (applied to individuals with AAD and their matched controls). Table S3: ATC‐codes prescriptions for treatment of osteoporosis (applied to individuals with AAD and their matched controls). Table S4: ICD‐10 codes of comorbidities related to increased fracture risk (applied to individuals with AAD and their matched controls). Table S5: Incident events of first major osteoporotic fracture among study participants. Table S6: Events of treatment with osteoporosis medication during the study period among study participants. Table S7: Sensitivity analysis on the effect of subgroups of oestrogen treatment (in women only) on the risk of MOF (applied to both individuals with AAD and controls). Table S8: Sensitivity analysis on the risk of MOF excluding individuals (patients with AAD and their matched controls) with ≥ 2 prescriptions of prednisolone (ATC‐code: H02AB06) before the start of followup. Table S9: Sensitivity analysis on the risk of MOF excluding individuals (patients with AAD and their matched controls) with a history of coeliac disease* before the start of follow‐up. Table S10: Sensitivity analysis on the risk of MOF adjusting for at least one dispensation of antihypertensive drugs* before the start of follow#x02010;up. Table S11: Sensitivity analysis on the risk of MOF for patients with incident diagnosis of AAD and their matched controls. Figure S1: Cumulative hazard plot of incident patients with AAD and their matched controls (incident cohort) with MOF [file JOIM-297-518-s001.docx]

Supplementary

Supplementary Table 1. Exclusion codes (applied to individuals with AAD and their matched controls).

| **Disease** | **ICD-10 1997-** | **ICD-9 1987-** | **ICD-8 1968-** | **ICD-7 1964-** |
| --- | --- | --- | --- | --- |
| Tuberculosis | A15-A19, P37.0, J65, B90 | 010–018, 137 | 010–019, Y34.09, Y34.19, Y34.29 | 001.99–019.20, Y03.00, Y03.10, Y03.20, Y53.00 |
| Waterhouse-Friedrichsens syndrome | A39.1 | 036D | 036.11 | 057.11 |
| HIV | B20–B24 | 079J, 279K |  |  |
| Malignant neoplasm, adrenal gland | C74, C79.7 | 194A, 198H | 194.01 | 195.01 |
| Benign neoplasm, adrenal gland | D35.0 | 227A | 226.00–09 | 224.10–224.19 |
| Neoplasm of uncertain or unknown behaviour, adrenal gland | D44.1 | 237C | 239.10 | 195.00, 239.31 |
| Idiopathic hypoparathyroidism | E20.0, E20.8, E20.9 | 252B, 252W, 252X | 252.10, 252.19, 252.98 | 271.10, 271.19, 271.29 |
| Adrenogenital disorders | E25 | 255C | 255.01, 255.02 |  |
| Drug-induced adrenocortical insufficiency | E27.3 |  |  |  |
| Other and unspecified adrenocortical insufficiency | E27.4 | 255F |  |  |
| Disorders of endocrine glands in diseases classified elsewhere | E35.1 | 255F |  |  |
| Disorders of fatty-acid metabolism (adrenoleukodystrophy) | E71.3 | 330A | 333.10 |  |
| Postprocedural adrenal hypofunction | E89.6 | 255F |  |  |
| Neonatal adrenal haemorrhage | P54.4 | 772F |  |  |
| Congenital malformations of adrenal gland | Q89.1 | 759B | 758.10 |  |
| Hyperfunction of pituitary gland | E22* | 253* | 253* | 272* |
| Hypofunction and other disorders of pituitary gland | E23* | 253* | 253* | 272* |
| Cushing syndrome | E24* | 255A | 258.00 | 277.10 |
| Malignant neoplasm, pituitary gland | C75.1 | 194D | 194.31 | 195.31 |
| Benign neoplasm, pituitary gland | D35.2 | 227D | 226.20 | 195.30, 224.40 |
| Neoplasm of uncertain or unknown behaviour, pituitary gland | D44.3 | 237A | 239.90 | 239.34 |
| Postprocedural hypopituitarism | E89.3 | 253H |  |  |

* All subcodes

Abbreviations: AAD: Autoimmune Addison’s disease, ICD: International classification of diseases, HIV: Human immunodeficiency viruses.

Supplementary Table 2. ICD-codes for fractures (applied to individuals with AAD and their matched controls).

| **Diagnosis** | **ICD-8** | **ICD-9** | **ICD-10** |
| --- | --- | --- | --- |
| Any MOF | 805, 806, 812, 813.00, 813.10, 813.90, 813.42, 813.52, 813.92, 820 | 805, 806, 812, 813E, 820 | S12, S22.0, S22.1, S32.0, S32.7, S42.2, S42.3, S42.4, S42.7, S52.5, S52.6, S72.0, S72.1, S72.2, T02.1, T08 |
| Any NVOF | 812, 813.00, 813.10, 813.90, 813.42, 813.52, 813.92, 820 | 812, 813E, 820 | S42.2, S42.3, S42.4, S42.7, S52.5, S52.6, S72.0, S72.1, S72.2 |
| Fracture of spine | 805, 806 | 805, 806 | S12, S22.0, S22.1, S32.0, S32.7, T02.1, T08 |
| Fracture of humerus | 812 | 812 | S42.2, S42.3, S42.4, S42.7 |
| Fracture of hip | 820 | 820 | S72.0, S72.1, S72.2 |
| Fracture of lower end of radius | 813.00, 813.10, 813.90, 813.42, 813.52, 813.92 | 813E | S52.5, S52.6 |

Abbreviations: AAD: Autoimmune Addison’s disease, MOF: Major osteoporotic fractures, NVOF: Non-vertebral osteoporotic fractures, ICD: International classification of diseases.

Supplementary Table 3. ATC-codes prescriptions for treatment of osteoporosis (applied to individuals with AAD and their matched controls).

| **ATC-code** | **Active substance** |
| --- | --- |
| M05BA01 | Etidronate |
| M05BB01 | Etidronate and calcium |
| M05BA02 | Clodronic acid |
| M05BA03 | Pamidronic acid |
| M05BA04 | Alendronic acid |
| M05BB03 | Alendronic acid and cholecalciferol |
| M05BB05 | Alendronic acid, calcium and cholecalciferol |
| M05BB06 | Alendronic acid and alfakalcidol |
| M05BA05 | Tiludronic acid |
| M05BA06 | Ibandronic acid |
| M05BA07 | Risedronic acid |
| M05BB02 | Risedronic acid and calcium |
| M05BB04 | Risedronic acid, calcium and cholecalciferol |
| M05BB07 | Risedronic acid and cholecalciferol |
| M05BA08 | Zoledronic acid |
| M05BB08 | Zoledronic acid, calcium and cholecalciferol |
| M05BX03 | Strontium ranelate |
| M05BX53 | Strontium ranelate and cholecalciferol |
| M05BX04 | Denosumab |
| M05BX06 | Romosozumab |
| H05AA02 | Teriparatide |
| H05AA04 | Abaloparatide |
| G03XC01 | Raloxifene |

Abbreviations: AAD: Autoimmune Addison’s disease, ATC: Anatomical Therapeutic Chemical Classification System.

Supplementary Table 4. ICD-10 codes of comorbidities related to increased fracture risk (applied to individuals with AAD and their matched controls).

| **Comorbidities** | **ICD-10** | **ATC** |
| --- | --- | --- |
| Type 1 diabetes | E10* |  |
| Type 2 diabetes | E11* |  |
| Malignant neoplasms | C00-96* |  |
| Thyrotoxicosis | E05* |  |
| Ischemic heart diseases | I20-I25* |  |
| Cerebrovascular diseases | I60-I69* |  |
| Chronic kidney disease | N18* |  |
| Diseases of liver | K70-K77* |  |
| Rheumatoid arthritis | M05-M06* |  |
| Testicular hypofunction | E29.1 |  |
| Primary ovarian failure | E28.3 |  |
| Chronic obstructive pulmonary disease (COPD) | J44* | N07BA** |
| Alcohol related disorders | F10* | N07BB*** |

* All subcodes. ** Drugs used in nicotine dependence. *** Drugs used in alcohol dependence.

Abbreviations: AAD: Autoimmune Addison’s disease, ICD-10: International classification of diseases version 10, ATC: Anatomical Therapeutic Chemical Classification System.

Supplementary Table 5. Incident events of first major osteoporotic fracture among study participants.

| **MOF** | **ICD-10** | **AAD n (%)** | **Controls n (%)** | **p-value*** |
| --- | --- | --- | --- | --- |
| Fracture of spine | S12, S22.0, S22.1, S32.0, S32.7, T02.1, T08 | 11 (14.3) | 64 (16.5) | 0,463 |
| Fracture of humerus | S42.2, S42.3, S42.4, S42.7 | 21 (27.3) | 75 (19.4) |  |
| Fracture of hip | S72.0, S72.1, S72.2 | 18 (23.4) | 106 (27.4) |  |
| Fracture of lower end of radius | S52.5, S52.6 | 27 (35.1) | 142 (36.7) |  |
| Total |  | 77 (100) | 387 (100) |  |

*Chi-squared test

Abbreviations: MOF: Major osteoporotic fractures, ICD-10: International classification of diseases version 10, AAD: Autoimmune Addison’s disease.

Supplementary Table 6. Events of treatment with osteoporosis medication during the study period among study participants.

| **Treatment of osteoporosis** | **AAD n (%)** | **Controls n (%)** | **p-value*** |
| --- | --- | --- | --- |
| Antiresorptive | 202 (98.5) | 574 (97.8) | 0.773 |
| Osteoanabolic | 1 (0.5) | 4 (0.7) | 1.000 |
| Other** | 2 (1.0) | 9 (1.5) | 1.000 |
| Total | 205 (100.0) | 587 (100.0) |  |

*Fisher’s exact test. ** Selective Oestrogen Receptor Modulators (SERM) and Strontium ranelate.

Abbreviations: AAD: Autoimmune Addison’s disease.

Supplementary Table 7. Sensitivity analysis on the effect of subgroups of oestrogen treatment (in women only) on the risk of MOF (applied to both individuals with AAD and controls).

| **Subgroup** | **Comparison group** | **Unadjusted Hazard Ratio (95% CI), p-value** | **Adjusted* Hazard Ratio (95% CI), p-value** | **p-value**  **for interaction** |
| --- | --- | --- | --- | --- |
| ≥ 2 oestrogen** dispensations before the start of follow-up | AAD, n=264 (26.0%) *** | 1.32 (0.75–2.30), p= 0.338 | 1.44 (0.72–2.91), p=0.302 | 0.256 |
|  | Controls, n=1470 (16.1%) *** | Reference | Reference |  |
| <2 oestrogen dispensations before the start of follow-up | AAD, n=750 (74.0%) | 1.87 (1.36–2.57), p<0.001 | 1.82 (1.29–2.57), p=0.001 |  |
|  | Controls, n=7639 (83.9%) | Reference | Reference |  |

* Adjusted for comorbidities (Table 1) and age at start of follow-up. ** ATC-codes: G03C: oestrogens (all subcodes) and G03F: gestagens in combination with oestrogens (all subcodes). *** ≥ 2 oestrogen dispensations before the start of follow-up for patients with AAD and controls: standardised difference: -0.170, p<0.001.

Abbreviations: MOF: Major osteoporotic fractures, CI: Confidence Intervals, AAD: Autoimmune Addison’s disease.

Supplementary Table 8. Sensitivity analysis on the risk of MOF excluding individuals (patients with AAD and their matched controls) with ≥ 2 prescriptions of prednisolone (ATC-code: H02AB06) before the start of follow-up.

| **Group** | **Unadjusted Hazard Ratio (95% CI), p-value** | **Adjusted* Hazard Ratio (95% CI), p-value** |
| --- | --- | --- |
| AAD | 1.88 (1.47–2.41), p<0.001 | 1.75 (1.34–2.29), p<0.001 |
| Controls | Reference | Reference |

* Adjusted for comorbidities (Table 1) and age at start of follow-up.

Abbreviations: MOF: Major osteoporotic fractures, ATC: Anatomical Therapeutic Chemical Classification System, CI: Confidence Intervals, AAD: Autoimmune Addison’s disease.

Supplementary Table 9. Sensitivity analysis on the risk of MOF excluding individuals (patients with AAD and their matched controls) with a history of coeliac disease* before the start of follow-up.

| **Group** | **n (%)** | **Adjusted** Hazard Ratio (95% CI), p-value** |
| --- | --- | --- |
| AAD | 60 (3.2%) | 1.80 (1.39–2.33), p <0.001 |
| Controls | 52 (0.3%) | Reference |

*ICD-10: K90.0, all subcodes. ** Adjusted for comorbidities (Table 1) and age at start of follow-up.

Abbreviations: MOF: Major osteoporotic fractures, AAD: Autoimmune Addison’s disease, CI: Confidence Intervals, ICD-10: International classification of diseases version 10.

Supplementary Table 10. Sensitivity analysis on the risk of MOF adjusting for at least one dispensation of anti-hypertensive drugs* before the start of follow-up.

| **Group** | **Adjusted** Hazard Ratio (95% CI), p-value** |
| --- | --- |
| AAD | 1.82 (1.41–2.35), p<0.001 |
| Controls | Reference |

* ATC-codes: C02, C03, C07, C08, C09 (all subcodes). ** Adjusted for comorbidities (Table 1), age at start of follow-up and one or more dispensations of antihypertensives before the start of follow-up.

Abbreviations: MOF: Major osteoporotic fractures, AAD: Autoimmune Addison’s disease, CI: Confidence Intervals, ATC: Anatomical Therapeutic Chemical Classification System.

Supplementary Table 11. Sensitivity analysis on the risk of MOF for patients with incident diagnosis of AAD and their matched controls.

| **Characteristic** | **AAD**  (n=893) | **Controls**  (n=8,572) |
| --- | --- | --- |
| Events of MOF, n | 27 | 119 |
| Median follow-up time (IQR), years | 4.49 (1.74–8.48) | 4.12 (1.13–8.57) |
|  | | |
| **Group** | **Unadjusted Hazard Ratio (95% CI), p-value** | **Adjusted* Hazard Ratio (95% CI), p-value** |
| Cases | 2.11 (1.41–3.15), p<0.001 | 2.18 (1.15–3.99), p=0.011 |
| Controls | Reference | Reference |
| Hydrocortisone dose (time varying variable) | 0.98 (0.88–1.09), p=0.739 | 1.01 (0.93–1.11), p=0.618 |
| Fludrocortisone dose (time varying variable) | 1.00 (0.98–1.05), p=0.148 | 1.01 (0.98–1.05), p=0.329 |

* Adjusted for time-varying hydrocortisone dose, time-varying fludrocortisone dose, comorbidities (Table 1), and age at start of follow-up.

Abbreviations: MOF: Major osteoporotic fractures, AAD: Autoimmune Addison’s disease, IQR: Interquartile range, CI: Confidence Intervals.

Supplementary Figure 1. Cumulative hazard plot of incident patients with AAD and their matched controls (incident cohort) with MOF


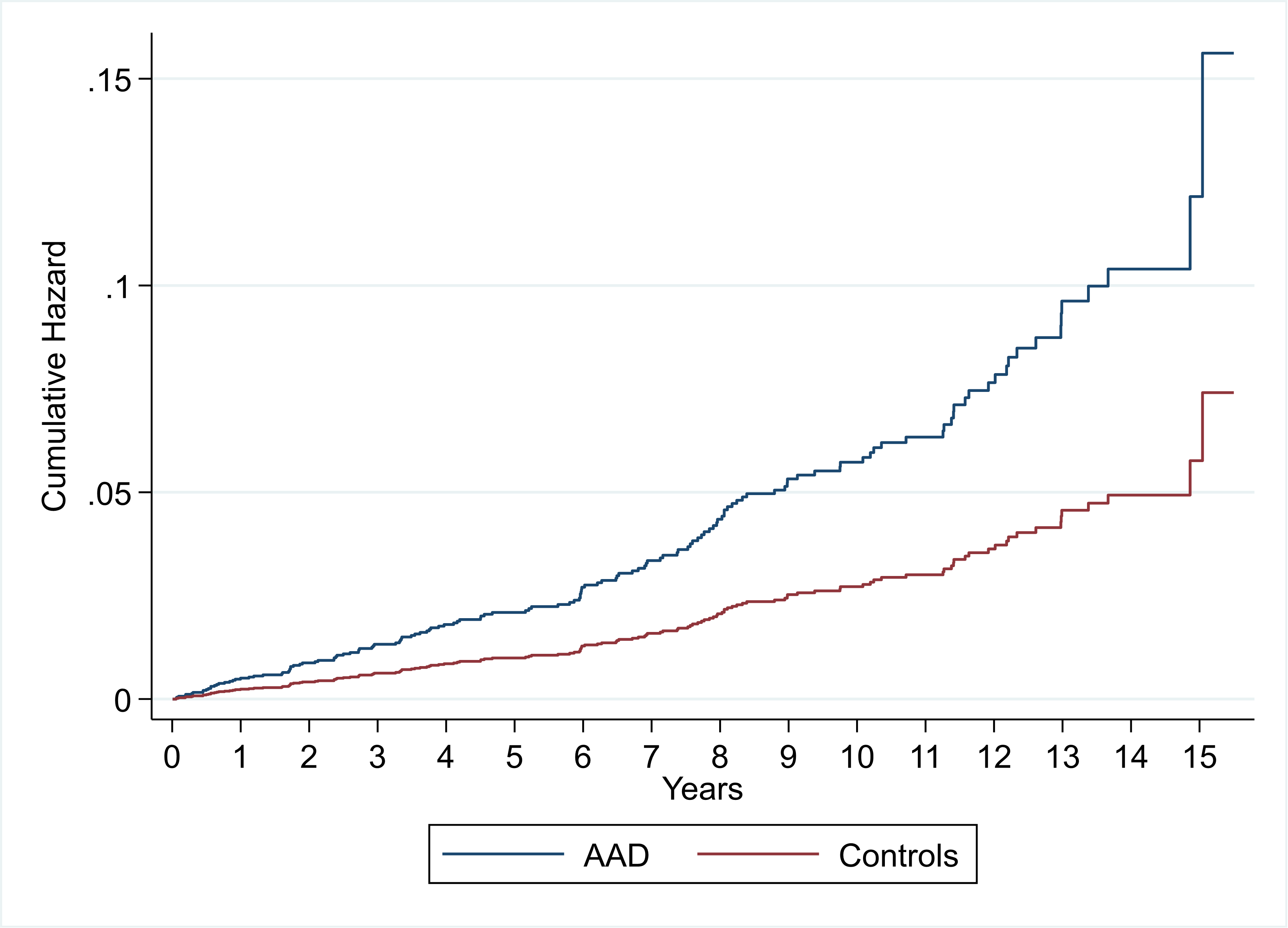


Abbreviations: AAD: Autoimmune Addison’s disease, MOF: Major osteoporotic fractures.
